# Vitamin D status and its determinants in healthy pregnant women living in Switzerland in the first trimester of pregnancy

**DOI:** 10.1186/s12884-018-2150-1

**Published:** 2019-01-08

**Authors:** Sophie Cabaset, Jean-Philippe Krieger, Aline Richard, Magdeldin Elgizouli, Alexandra Nieters, Sabine Rohrmann, Katharina C. Quack Lötscher

**Affiliations:** 1 Epidemiology, Biostatistics and Prevention Institute, Division of Chronic Disease Epidemiology, University of Zurich, Hirschengraben 84, CH-8001 Zurich, Switzerland; 2 Center for Chronic Immunodeficiency, Medical Center - University of Freiburg, Faculty of Medicine, University of Freiburg, Breisacherstr. 115 4, 79106 Freiburg, Germany; 3 Clinic of Obstetrics, University Hospital Zurich, Frauenklinikstrasse 10, CH-8091 Zurich, Switzerland

**Keywords:** 25-hydroxy-vitamin D, Country of origin, Smoking status, Supplements, Pregnancy

## Abstract

**Objectives:**

Our study aimed at assessing the prevalence and determinants of vitamin D deficiency (25-hydroxy-vitamin D [25(OH)D] < 20 ng/mL) in pregnant women in the first trimester living in Switzerland.

**Methods:**

From September 2014 through December 2015, 204 pregnant women were conveniently recruited during their first clinical appointment at the Clinic of Obstetrics of the University Hospital Zurich (between week 6 and 12 of pregnancy). Blood samples were collected and a questionnaire focusing on lifestyle and skin colour was completed face-to-face with the responsible physician. Logistic regression analyses were performed with vitamin D status as dependent variable.

**Results:**

63.2% of the participating women were vitamin D deficient, and the median vitamin D concentration in the overall sample was 17.1 ng/mL [Q1, Q3: 9.78, 22.3]. The highest proportions of vitamin D deficiency were detected in women originating from Africa and Middle East (91.4% deficient, median vitamin D concentration of 10.7 ng/mL [Q1, Q3: 6.55, 14.45]) and from South-East Asia/Pacific (88.5% deficient, median vitamin D concentration of 8.4 ng/mL [Q1, Q3: 6.10, 14.88]). Multivariable logistic regression showed that significant risk factors of vitamin D deficiency were country of origin (women born in Switzerland and Germany had a lower risk than women born in other countries), smoking status (lower risk for former smokers) and intake of vitamin D supplements.

**Conclusions:**

Our results confirm a high prevalence of vitamin D deficiency in this Swiss cohort, in particular in women coming from Asian and African countries, and underline the importance of appropriate counseling and vitamin D supplementation in early pregnancy.

**Electronic supplementary material:**

The online version of this article (10.1186/s12884-018-2150-1) contains supplementary material, which is available to authorized users.

## Introduction

Vitamin D is a fat-soluble vitamin acknowledged for its importance in maintaining bone health. Suboptimal vitamin D levels have also been associated with higher frequencies of different types of cancers [1–3], cardiovascular diseases [1] and auto immune diseases such as multiple sclerosis [4, 5], rheumatoid arthritis [6] and type-1 diabetes [1, 7].

Vitamin D occurs naturally in a limited number of foods but is mainly synthesized by the skin through UVB light exposure [8]. After ingestion or cutaneous formation, vitamin D is first converted by the liver to 25-hydroxy-vitamin D (25(OH)D) and then to 1,25-dihydroxyvitamin D (1,25(OH)_2_D) in the kidney. Although1,25(OH)_2_D is vitamin D’s biologically active form, vitamin D status is commonly determined by measuring serum concentration of 25(OH)D.

Most humans depend on sun exposure to satisfy their requirements for vitamin D, but in countries above latitude 35°North (and South), UVB radiation is insufficient to enable endogenous vitamin D production all year round [9]. Since Switzerland is at a latitude of 46°N a large percentage of the Swiss population is likely to be vitamin D deficient [10]. This is particularly true during winter months when sun exposure and UVB radiations are inadequate for cutaneous vitamin D formation [11]. Numerous studies have reported associations between vitamin D deficiency during pregnancy and adverse outcomes both on maternal health and fetal development [12]. Poor maternal vitamin D status has indeed been correlated with pregnancy complications such as preeclampsia, premature birth, infants born small for gestational age [13–16] and respiratory tract infections among children [17]. According to a report issued in 2012, the vitamin D status, and consequently the prevalence of vitamin D deficiency in pregnant women living in Switzerland remains mostly unknown [18]. Even if most of the reported determinants of vitamin D deficiency are similar to the ones reported for the general population, some determinants seem to remain sub-group- and country-specific [19]. The understanding of vitamin D status in early pregnancy and the importance of the role of the country of origin and other predictors to explain this deficiency may help to reduce health inequalities among women living in Switzerland and guide future public health policies. More generally, our research will contribute to increasing our knowledge on the vitamin D status of pregnant women in the Swiss population.

## Methods

### Study design

The study design was described elsewhere [20]. In summary, pregnant women in their first trimester of pregnancy (between week 6 and 12 of pregnancy) were conveniently recruited between September 2014 through December 2015 while attending their first routine antenatal care appointment at the Clinic of Obstetrics of the University Hospital Zurich (USZ). Inclusion criteria were pregnancy, plans to deliver at the Clinic of Obstetrics of the USZ, minimum age of 18 years, current residence in Switzerland for at least 6 months before the start of pregnancy, and fluency in German, French, Italian or English. Exclusion criteria were multiple pregnancies, HIV infection, history of parathyroid, renal or liver diseases, chronic malabsorption syndromes or granuloma-forming disorders, age below 17 years or known (or suspected) drug or alcohol abuse. For eligible participants, an informed consent was obtained by the physician. A 10 mL blood sample was drawn during routine blood collection. Study participants and the responsible physician completed together a questionnaire (see Additional file 1) aiming at gathering socio-demographic information as well as data related to the pregnancy and lifestyle. Our final sample consisted of 204 women. The study was not representative of the whole Swiss population, but the choice of the USZ was driven by the heterogeneity in the socio-demographic and cultural background of women attending this hospital. In general, the city of Zurich has a large foreign population (32%) [21]. A true sample size calculation was not available in practice, because the coefficients of determination between covariates could not be determined. However, a minimum of ten participants per covariate were included in the analysis, as suggested by Agresti [22] for multivariable logistic regressions.

Approval (KEK-ZH-Nr. 2013–0213) was provided by the cantonal ethics committee of Zurich, Switzerland.

### Laboratory analyses

The Institute of Clinical Chemistry of the University Hospital Zurich analysed all blood samples within hours following collection: after centrifugation and serum extraction, total 25-hydroxyvitamin D (25(OH)D) was analyzed with the vitamin D total-analysis Roche Cobas® electro-chemo-luminescence immunoassay (Roche Diagnostics, Basel, Switzerland; detection range: 3.0–70.0 ng/mL for 25(OH)D; above 15 ng/mL, inter-assay coefficient of variation: 11.5% and intra-assay coefficient: 6.5% [23]). Women with serum 25(OH)D concentrations strictly below 20 ng/mL were considered as vitamin D deficient, as recommended by the Endocrine Society [24]. Women with serum 25(OH)D concentrations above 20 ng/mL were considered as non-deficient (which includes both insufficient (20 to 30 ng/mL) and sufficient (above 30 ng/mL) as defined by the Endocrine Society [24]).

### Variables and determinants of vitamin D status

The variables and potential determinants of vitamin D status were retrieved from the questionnaire (Additional file 1) and medical records. In summary, we collected age, week of pregnancy, parity (nulliparous yes, no), gravidity or first pregnancy (yes, no), Body Mass Index (BMI) before pregnancy (self-reported), BMI at enrolment (self-reported, mentioned in tables as BMI current), skin colour type, country of origin categorized into five groups, education level of the mother and of the partner in line with the International Standard Classification of Education [20] (less than compulsory education and compulsory education; secondary education; tertiary education), smoking status (never, ever, current), season at enrolment (winter [December 21st – March 20th], spring [March 21st – June 20th], summer [June 21st – September 20th], autumn [September 21st – December 20th]), sun exposure as the average number of days per week spent at least 1 h outdoor between 10 am and 4 pm in the past 6 months, use of sun protection (such as use of sunscreen, wearing long sleeves, trousers, hat (never, sometimes, always), fish consumption (herring, salmon, mackerel, sardine or tuna) at least once a week (yes, no) and intake of vitamin supplements containing vitamin D (yes, no). Vitamin D containing supplements were either pure vitamin D supplements or multivitamin supplements containing vitamin D. Dosage, adherence to treatment and start of intake were not recorded. We grouped countries of origin in five categories based on the regions defined by the world National Bank and previously reported [20] (group 1: Switzerland and Germany; group 2: Northern America, Northern Europe, Central Asia and New-Zealand; group 3: Southern Europe, Australia and Latin America; group 4: South-East Asia Pacific; group 5: Africa and Middle East). We have previously shown that dark skin colour was associated with a higher prevalence of vitamin D deficiency [20]. Eventually, we decided to assess vitamin D concentrations under the angle of the country of origin and compare the differences between subgroups. Country of origin is informative and important in clinical practice as it encompasses aspects such cultural behaviours, physical activity, eating habits and attitude towards sun exposure in addition to skin colour. We then proceeded to identify the significant determinants of vitamin D deficiency in early pregnancy and finally established a prospective prediction model. Several models were compared with the objective to identify the best compromise between statistical validity, ease of data collection and practicality in a clinical environment.

Women’s skin colour was assessed according to the classification by Fitzpatrick [25] using a five-level scale [20]. The classification into skin types was assessed in two steps. In a first step, study participants were shown a picture of the different skin colour types (I-V) and rated their own type. The same evaluation was done in parallel by the physician. In a second step, women were asked to classify in one of five predefined categories what happens to their untanned skin when exposed to the sun under specific conditions [20]. Based on their skin colour type and skin tanning evaluations, women estimated their own skin phototypes. When the classification of a pregnant woman and the interviewer disagreed, the upper rounded arithmetic mean was used to determine the skin colour type.

Additionally, we defined a melanin index [26] on the basis of measurements conducted with a narrow band spectroscopy instrument (DSM II ColourMeter, Cortex Technology, Hadsund, Denmark; green diode 568 nm, red diode 655 nm). Measurements were repeated 3 times on the inner underarm and the arithmetic mean was calculated. The instrument was calibrated on a weekly basis using a white balance.

### Statistical analyses

Statistical analyses and graphing were carried out using R version 3.3.2 for Windows. Boxplots represent the medians and first and third quartiles. Prior to starting statistical analyses, categorical variables, for which the size of certain subsets was insufficient to guarantee the strength of statistical testing, were transformed and levels combined when deemed necessary. Skin colour phototypes were dichotomized into light skin colour (Fitzpatrick scale I to III) vs. dark skin colour (Fitzpatrick scale IV and V). For the variables education level of the mother and education level of the partner, individuals with “less than compulsory education” and “compulsory education” were grouped into one level (level 1). Finally, melanin index was taken into account both as a continuous variable and split into tertiles.

Univariable logistic regression analyses were performed with vitamin D status (deficient versus non-deficient) as dependent variable to estimate associations between vitamin D deficiency and following potential determinants: age, week of pregnancy, nulliparity, gravidity, BMI before pregnancy, BMI at enrolment, skin colour, country of origin, education level achieved by the mother and by the partner, smoking status, season, sun exposure, use of sun protection, fish consumption and intake of vitamin D-containing supplements. Multivariable logistic regressions with vitamin D status (deficient versus non-deficient) as dependent variable were subsequently performed. The Akaike information criterion (AIC) score and the area under the curve of the receiver operating characteristics (c-index) and likelihood ratio test Chi-square test (LRT) were used for selecting the final model. Calibration curves were realized to display how much the predictive values calculated with our models differed from the observed values. These curves were obtained by binning our data by halves: data was first split in upper and lower half, these halves were in their turn split in halves and finally, extreme halves were split recursively. Both predictive and observed values were binned and averaged within each bin. Dots sizes are proportional to the number of observations within a bin.

Model 1, also referred to as full model, included all probable determinants, excluding variables highly associated with each other. Associations between variables were tested using Cramér’s V for categorical and Pearson’s correlation coefficient for continuous variables. Model 2 was the most parsimonious model. Finally, model 3 was determined using backward-forward AIC selection principles. For all models, the measure of association was the odds ratio (OR) and its corresponding 95% confidence interval (95% CI). *P* value threshold was set at *p* < 0.05. The goodness of fit and best prediction power assessments were performed by comparing the c-statistics of all three models.

## Results

### Characteristics of the studied population

Among the 204 pregnant women in their first trimester participating in the study (Table 1), 22.1% originated from Switzerland or Germany (group 1), 34.3% from North America, Northern Europe, Caucasus, Central Asia and New Zealand, excluding Germany and Switzerland (group 2), 13.7% from Southern Europe, Australia, Latin America and the Caribbean (group 3), 12.7% from South East Asia Pacific (group 4) and 17.2% from Africa and Middle East (group 5). The median vitamin D concentration in the overall sample was 17.1 ng/mL with 63.2% of participating women being vitamin D deficient. The highest proportions of vitamin D deficiency were detected in group 4 (88.5% of deficiency, median vitamin D concentration of 8.4 ng/mL) and group 5 (91.4% of deficiency, median vitamin D concentration of 10.7 ng/mL). The overall mean age at blood collection was 30 years, with women in group 1 being the oldest. All women in group 1 were fair skinned, whereas more than 65% of women in groups 4 and 5 were dark skinned. The highest proportion of pregnant women with an education level less than compulsory and compulsory was observed in group 5 (54.3%). More than half of the women in the overall sample had never smoked with the highest proportion of never and former smokers seen in groups 4 and 5. 75% of women ate fish more than once a week, which was similar among the different subsets. Finally, 37.8% of women used vitamin D supplements, with the highest proportion in group 3 (53.6%).

**Table 1:** General characteristics of study participants by country of origin (*n* = 204)

	Overall^g^	Switzerland and Germany	North America, Northern Europe, Caucasus, Central Asia and New zealand (excluding Switzerland and Germany)	Southern Europe, Australia, Latin America and the Caribbean	South East Asia Pacific	Africa and Middle East
n (% of total women included)	204	45 (22.05)	70 (34.31)	28 (13.73)	26 (12.75)	35 (17.16)
Mothers, VitD deficient^a^, n (%)	129 (63.24)	18 (40.00)	42 (60.00)	14 (50.00)	23 (88.46)	32 (91.43)
Mothers, 25(OH)D ng/mL, median (Q1,Q3)	17.10 (9.78–22.30)	21.40 (17.30–25.60)	17.85 (9.98–26.13)	19.95 (12.95–22.10)	8.40 (6.10–14.88)	10.70 (6.55–14.45)
Age, mean (SD)	30.03 (4.85)	32.18 (4.44)	30.04 (5.06)	29.68 (5.06)	27.92 (4.02)	29.09 (4.51)
Week of pregnancy, median (Q1, Q3)	8 (7.75–9)	8 (7–9)	8 (7–9)	8 (8–9)	8 (8–9)	9 (8–11.5)
Nulliparity, n (%)	108 (52.94)	27 (60.00)	36 (51.43)	17 (60.71)	15 (57.69)	13 (37.14)
First pregnancy, n (%)	84 (41.18)	19 (42.22)	28 (40.00)	12 (42.86)	15 (57.69)	10 (28.57)
BMI before pregnancy (kg/m^2^), median (Q1, Q3)	21.82 (19.93–24.78)	21.30 (19.72–23.19)	20.96 (19.74–23.45)	21.76 (20.32–25.12)	22.77 (20.00–24.72)	24.48 (22.01–27.26)
BMI current (kg/m^2^), median (Q1, Q3)	22.20 (20.42–25.67)	21.97 (20.37–23.51)	21.56 (20.29–23.81)	22.55 (20.79–26.59)	23.09 (20.18–25.36)	25.75 (21.44–28.09)
Melanin index, median (Q1, Q3)	33.59 (30.82–39.57)	32.34 (30.48–33.46)	31.78 (29.90–34.04)	37.27 (34.09–41.19)	43.39 (34.34–46.04)	42.22 (34.23–56.73)
Melanin index Group 1^b^, n(%)	68 (33.33)	18 (40.00)	36 (51.43)	4 (14.28)	2 (7.69)	8 (22.86)
Melanin index Group 2^b^, n(%)	68 (33.33)	22 (48.89)	26 (37.14)	9 (32.14)	6 (23.08)	5 (14.29)
Melanin index Group 3^b^, n(%)	68 (33.33)	5 (11.11)	8 (11.43)	15 (53.57)	18 (69.23)	22 (62.85)
Skin colour, n(%)						
Light^c^	151 (74.02)	45 (100)	66 (94.29)	20 (71.43)	8 (30.79)	12 (34.29)
Dark^d^	53 (25.98)	0	4 (5.71)	8 (28.57)	18 (69.21)	23 (65.71)
Education^e^, n (%)						
Level 1	37 (18.14)	1 (2.22)	7 (10.00)	7 (25)	3 (11.54)	19 (54.29)
Level 2	68 (33.33)	21 (46.67)	17 (24.29)	12 (42.86)	6 (23.08)	12 (34.28)
Level 3	99 (48.53)	23 (51.11)	46 (65.71)	9 (32.14)	17 (65.38)	4 (11.43)
Education of the partner ^e^, n (%)						
Level 1	33 (16.17)	3 (6.67)	4 (5.71)	4 (14.29)	3 (11.54)	19 (54.29)
Level 2	79 (38.73)	19 (42.22)	24 (34.29)	14 (50.00)	9 (34.62)	13 (37.14)
Level 3	90 (44.12)	23 (51.11)	40 (57.14)	10 (35.71)	14 (53.85)	3 (8.57)
Smoking status, n (%)						
Never	121 (59.31)	15 (33.33)	40 (57.14)	15 (53.57)	22 (84.62)	29 (82.86)
Ever	62 (30.39)	21 (46.67)	24 (34.29)	8 (28.57)	4 (15.38)	5 (14.29)
Current	21 (10.30)	9 (20.00)	6 (8.57)	5 (17.86)	0	1 (2.86)
Season, n (%)						
Winter	65 (31.86)	14 (31.11)	23 (32.86)	12 (42.86)	8 (30.77)	8 (22.86)
Spring	46 (22.55)	10 (22.22)	14 (20.00)	4 (14.29)	4 (15.38)	8 (22.86)
Summer	53 (25.98)	23 (28.89)	14 (20.00)	10 (35.71)	4 (15.38)	12 (34.28)
Fall	40 (19.61)	8 (17.78)	19 (27.14)	2 (7.14)	10 (38.47)	7 (20.00)
Days per week spent at least 1 h outdoor in the past 6 months, median (Q1, Q3)	2 (2–7)	2 (2–4)	2 (2–7)	2 (2–6.25)	7 (2–7)	3 (1–7)
Using sun protection in summer, n (%)						
Never	50 (24.51)	2 (4.44)	13 (18.57)	10 (35.71)	12 (46.15)	13 (37.14)
Sometimes	78 (38.24)	19 (42.22)	39 (55.71)	9 (32.14)	7 (26.92)	4 (11.43)
Always	75 (36.74)	24 (53.33)	18 (25.71)	9 (32.14)	7 (26.92)	17 (48.57)
Fish consumption at least once a week, n (%)	151 (74.02)	33 (73.33)	55 (78.57)	21 (75.00)	19 (73.08)	23 (65.71)
Vitamin D supplement intake, n (%)^f^	77 (37.75)	16 (35.56)	24 (34.29)	15 (53.57)	10 (38.46)	12 (34.29)

^a^25(OH)D < 20 ng/mL

^b^Melanin index: *Group 1* = values between 16.6–31.9; *Group 2* = values between 31.9–37.1; *Group 3* = values between 37.1–68.00

^c^Light skin colour defined as value I to III from the Fitz Patrick scale

^d^Dark skin colour defined as value IV to V from the Fitz Patrick scale

^e^*Level 1* = Less than compulsory education and Compulsory education; *Level 2* = Secondary education; *Level 3* = Tertiary education

^f^All types of supplements containing vitamin D (multivitamin included)

^g^Information missing: BMI before pregnancy (*n* = 6), current BMI (*n* = 6), education of partner (*n* = 2), Days per week spent at least 1 h outdoor in the past 6 months (*n* = 2), using sun protection in the summer (*n* = 2), fish consumption (*n* = 2)

### Determinants of vitamin D deficiency in early pregnancy

Results obtained through univariable logistic regression analyses (Table 2) revealed that risk factors for vitamin D deficiency were higher BMI before pregnancy and at enrolment, higher melanin index, dark skin colour, and country of origin (women originating from country groups 2, 4, and 5 had higher odds of deficiency than group 1). Conversely, older age, education level of both the mother and her partner (the higher the education level the lower the odds of deficiency), smoking status (former smokers had a lower odds of low vitamin D circulating level), use of sun protection (women never or sometimes using sun protection suffered less from vitamin D deficiency) and the intake of vitamin D-containing supplements, had a protective effect.

**Table 2:** Odds of vitamin D deficiency early in pregnancy (*n* = 204)

	Univariable models		Multivariable model 1^g^		Multivariable model 2^h^		Multivariable model 3^i^	
	OR	95% CI	OR	95% CI	OR	95% CI	OR	95% CI
Age	0.93	0.87–0.99	0.98	0.90–1.06	–	–	–	–
Week of pregnancy	1.04	0.87–1.25	0.86	0.68–1.08	–	–	–	–
Nulliparity^a^	1.26	0.70–2.28	–	–	–	–	–	–
First pregnancy^b^	1.23	0.68–2.24	1.16	0.49–2.75	–	–	–	–
BMI before pregnancy	1.10	1.01–1.20	–	–	–	–	–	–
BMI current	1.10	1.02–1.21	1.08	0.98–1.20	1.08	0.99–1.19	–	–
Melanin index								
Melanin index as a continuous variable	1.04	1.00–1.08	–	–	–	–	–	–
Melanin index Group 1^c^	1.14	0.56–2.34	–	–	–	–	–	–
Melanin index Group 2^c^	1.00	*(ref)*	–	–	–	–	–	–
Melanin index Group 3^c^	2.01	0.96–4.27	–	–	–	–	–	–
Skin colour								
Light	1	*(ref)*	–	–	–	–	–	–
Dark	3.92	1.84–9.16	–	–	–	–	–	–
Country of origin^d^								
Group 1	1	*(ref)*	1	*(ref)*	1	*(ref)*	1	*(ref)*
Group 2	2.56	1.17–5.79	2.29	0.88–6.16	2.57	1.1–6.2	2.32	1.07–5.17
Group 3	1.45	0.54–3.92	1.07	0.32–3.51	1.57	0.55–4.54	1.85	0.69–5.06
Group 4	12.94	3.77–60.91	11.71	2.62–67.84	14.57	3.84–73.97	13.35	3.8–64.68
Group 5	15.75	4.65–73.58	9.38	2.12–54.11	14.48	3.93–71.71	17.89	5.24–84.42
Education ^e^								
Level 1	1	*(ref)*	1	*(ref)*	–	*–*	–	–
Level 2	0.31	0.10–0.80	0.64	0.17–2.26	–	*–*	–	–
Level 3	0.26	0.09–0.64	0.63	0.15–2.48	–	*–*	–	–
Education of the partner^e^								
Level 1	1	*(ref)*	–	–	–	–	–	–
Level 2	0.55	0.19–1.47	–	–	–	–	–	–
Level 3	0.24	0.08–0.62	–	–	–	–	–	–
Smoking status								
Never	1	*(ref)*	1	*(ref)*	1	*(ref)*	–	–
Ever	0.34	0.18–0.67	0.40	0.17–0.91	0.49	0.23–1.04	–	–
Current	0.86	0.31–2.64	1.88	0.64–7.23	2.65	0.88–8.91	–	–
Season								
Winter	1	*(ref)*	1	*(ref)*	–	*–*	–	–
Spring	1.36	0.57–3.35	1.23	0.42–3.65	–	*–*	–	–
Summer	0.57	0.26–1.24	0.51	0.20–1.31	–	*–*	–	–
Fall	0.93	0.41–2.13	0.88	0.32–2.42	–	*–*	–	–
Days per week spent at least 1 h outdoor in the past 6 months	1.07	0.95–1.20	0.95	0.81–1.11	–	*–*	–	–
Using sun protection in summer								
Never	1	*(ref)*	1	*(ref)*	–	*–*	–	–
Sometimes	0.27	0.11–0.61	0.51	0.17–1.47	–	*–*	–	–
Always	0.42	0.17–0.95	0.65	0.2–2.01	–	*–*	–	–
Fish consumption at least once a week	0.57	0.27–1.13	0.69	0.28–1.65	–	*–*	–	–
Vitamin D supplement intake^f^	0.44	0.24–0.80	0.30	0.14–0.63	0.33	0.16–0.65	0.37	0.19–0.71
Comparison of linear regression models: Goodness of fit and best prediction power assessments								
AIC			253.19		216.14		220.09	
c-index (95% CI)			0.821	(0.761–0.881)	0.796	(0.731–0.860)	0.765	(0.698–0.832)

^a^non nulliparous

^b^not the first pregnancy

^c^Melanin index: *Group 1* = values between 16.6–31.9; *Group 2* = values between 31.9–37.1; *Group 3* = values between 37.1–68.00

^d^*Group 1* = Switzerland and Germany; *Group 2* = North America, Northern Europe, Caucasus, Central Asia and New Zealand (excluding Switzerland and Germany); *Group 3* = Southern Europe, Australia, Latin America and the Caribbean; *Group 4* = South East Asia Pacific; *Group 5* = Africa and Middle East

^e^*Level 1* = Less than compulsory education and Compulsory education*; Level 2 =* Secondary education*; Level 3* = Tertiary education

^f^All types of supplements containing vitamin D (multivitamin included)

^g^Model 1: Full model - selection of categorical variables according to Cramer’s V correlation analysis. Selection of continuous variables according to Pearson’s correlation coefficient

^h^Model 2: model selected using AIC-based forward backward selection

^i^Model 3: Parsimonious model

Before proceeding to multivariable logistic regressions, we tested the collinearity between statistically significant variables. The following pairs of variables were considered as correlated: gravidity and nulliparity (Cramér’s V = 0.79), education of the mother and education of the partner (Cramér’s V = 0.56), skin colour and country of origin (Cramér’s V = 0.64), and finally BMI before pregnancy and BMI at enrolment (Pearson’s correlation coefficient = 0.98). Consequently, a decision was made to exclude the variables nulliparity, education of the partner, skin colour and BMI before pregnancy from further analyses as we considered them the least informative. As a result, the first model analyzed (model 1) included age, week of pregnancy, BMI at enrolment, gravidity, country of origin, education of the mother, smoking status, season, days spent in the sun, use of sun protection, fish consumption and use of vitamin D containing supplements.

**Fig. 1:**
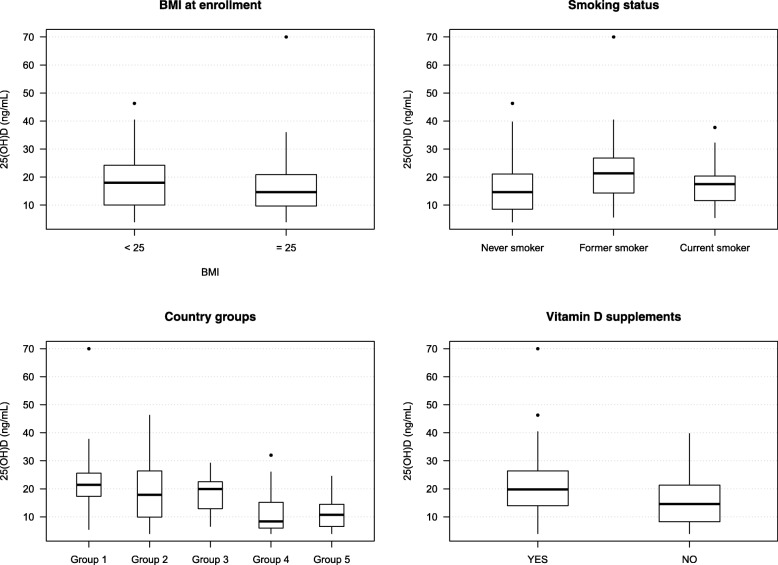
Serum 25(OH)D levels in pregnant women by BMI at enrolment, smoking status, country groups, and intake of vitamin D supplements. Boxplots represent the median, 1st and 3rd quartiles of the complete cases

**Fig. 2:**
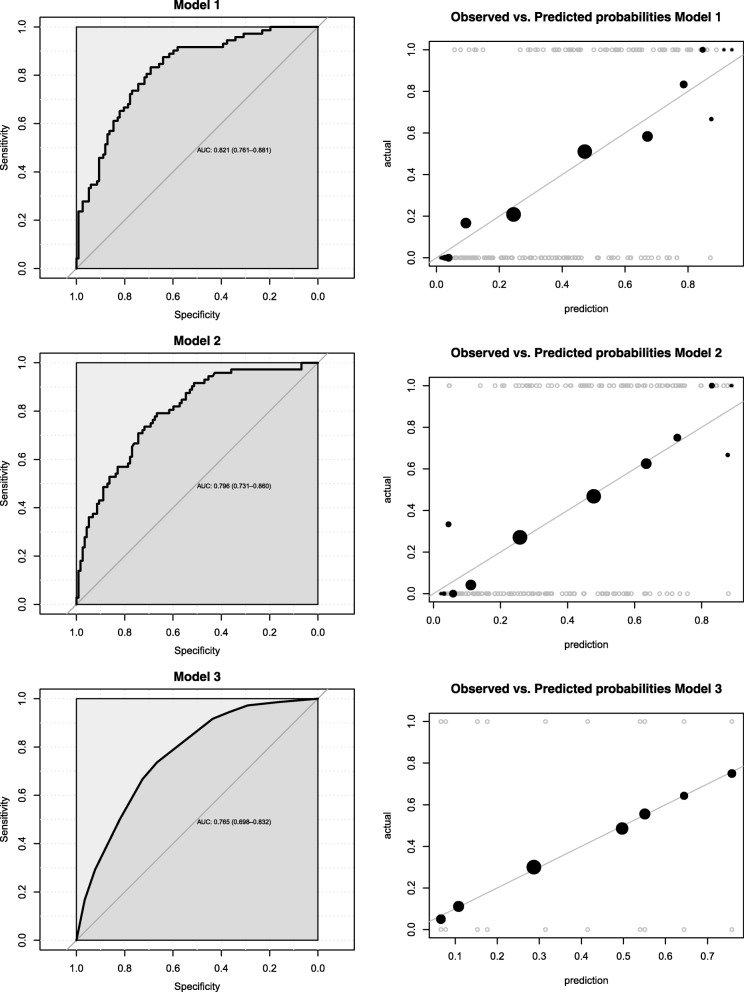
Receiver operating characteristic (ROC) curves and observed vs. predicted value plots for models 1, 2 and 3

Compared to the univariable logistic regressions results, country of origin, smoking status and use of vitamin D-containing supplements remained significant determinants of vitamin D deficiency, and BMI at enrolment remained close to significance (Model 1; Table 2). All other variables did not reach the significance level (*p* < 0.05). Concentrations of 25(OH)D across levels of the four statistically significant (or close to significant) determinants of vitamin D deficiency are displayed in Fig. 1. In Model 2, country of origin, BMI at enrolment, smoking status and use of vitamin D-containing supplements best explained vitamin D deficiency within this Swiss cohort. Model 3 was the most parsimonious model. Model 2, with an AUC of 0.796, a good calibration and an AIC value of 220.09 corresponded to the most easily adaptable model for practical prediction of Vitamin D deficiency (Fig. 2 and Table 2). Though it could be argued that model 3 as the most parsimonious model would better serve this purpose, LRT Chi-square tests between model 2 and 3 were performed. Results indicated that model 2 fits the data significantly better than model 3 (LRχ^2^: 10.46, DF: 3, *p* = 0.015) recommending the former as the best model to predict vitamin D deficiency in practice. While Receiver Operator Characteristic (ROC) curves supported the discrimination power assessment of the models, we used calibration curves to display the degree of accuracy of predictive values obtained with our models vs. observed values (Fig. 2).

## Discussion

Although numerous adverse outcomes have been associated with vitamin D deficiency, the public health prevention strategy to reduce vitamin D deficiency still seems inadequate in Switzerland. Our study, realized in 2014 and 2015, was the first one to assess risk factors for vitamin D deficiency in pregnant women living in Switzerland. The level of deficiency among pregnant women in their first trimester of pregnancy and living in Zurich attained almost two-thirds of the sample. This was a higher prevalence than those observed in some recent European and American studies (Additional file 2: Table S1 [27–31]). Similarly, the median 25(OH)D concentration in the present study (17.1 ng/mL) was lower than mean or median values reported (Additional file 2: Table S1 [27–31]). Likewise, results in our study of pregnant women living in Switzerland in their 3rd trimester of pregnancy had both a prevalence of vitamin D deficiency and a median 25(OH)D circulating level worse than in other European countries [32]. This result is likely to raise questions in a country like Switzerland with high standards of living, high health expenditure (11.4% of GDP in 2013 vs. OECD average of 8.9%) [33], and clear recommendations for daily vitamin D intake during pregnancy [34].

Our study population was very heterogeneous with only 22% of the women originating from Switzerland and Germany. But also in this subgroup, mean 25(OH)D level was only slightly above the threshold for vitamin D deficiency (21.4 ng/mL). All other subgroups had a median 25(OH)D level below 20 ng/mL. Women from South East Asia and from Africa and the Middle East in our sample had a greater risk of being vitamin D deficient than the rest of the studied population. A similar association had been observed in Belgian and Dutch studies, in which ethnicity and immigrant background were discussed as potential determinants of vitamin D deficiency during pregnancy [27, 35]. However, a Spanish study assessing country of birth as a predictor of 25(OH)D deficiency did not observe a significant association [29], which might be due to the small number of women originating from a foreign country.

Our results might be driven by a higher prevalence of dark-skinned individuals in South East Asian and African subsets. Skin colour was identified as a significant predictive factor in univariable logistic regression analysis, but it was no longer considered in the multivariable analysis as collinearity analysis confirmed a high correlation with the country of origin. However, skin colour remains an important factor, which should be taken into consideration when assessing vitamin D deficiency risk in a population. As a matter of fact, skin pigmentation, which is thought to have developed as an evolutionary protective adaptation from the damaging effects of excessive direct sun exposure [36], has been identified as a predictor of vitamin D deficiency. Humans with dark skin pigmentation have skin naturally rich in melanin (especially eumelanin). Melanin functions as a shield and absorbs ultraviolet B radiations reducing vitamin D3 synthesis by > 90% [37]. Therefore, dark-skinned individuals need longer sunshine exposure in order to produce the same amount of vitamin D than individuals with less pigmented skins. People moving to areas of reduced ultraviolet B radiations compared to their home countries might be at higher risk of vitamin D deficiency. This situation could be further complicated by cultural habits such as traditional skin covering (veiling), vitamin D-poor diets and avoidance of direct sun exposure, which has also been shown in a review of vitamin D deficiency in Mediterranean countries [38].

Smoking habits were another lifestyle factor identified as significantly impacting the vitamin D status of pregnant women in our study. Interestingly, women who had quit smoking had lower odds of being vitamin D deficient than never-smokers. We do not believe that being a former smoker has a protective effect with respect to vitamin D deficiency. More likely, former smokers, especially women who quit smoking for a pregnancy, might have a different, more conscious health behaviour than other women. Although a Danish survey of healthy women aged 45–58 years revealed that smokers had significantly reduced levels of serum 25(OH)D [39], evidence for pregnancy remains controversial. In a Belgian study, smoking was established as increasing the risk of vitamin D deficiency in pregnancy [27]. A Spanish study conducted during the perinatal period showed identical patterns revealing that serum 25(OH)D levels were higher in women who continued smoking while pregnant than in non-smoking pregnant women [40]. Yet, another Spanish study did not observe any significant relationship between smoking habits and Vitamin D deficiency during early pregnancy [41].

Vitamin D supplement intake was a predictor of vitamin sufficiency in our sample. In a British study, 25(OH)D levels in pregnant women were significantly higher in women randomized to vitamin D supplementation than to placebo. However, despite supplementation, 20% of the recruited women did not achieve repletion [42]. Similar effects were observed in an intervention performed in the northern part of the United States, where black women, even though compliant with prenatal supplement use remained at high risk of vitamin D insufficiency. One of this study’s limitations, however, was the lack of information on supplement dosages [43]. In our sample, of the 129 women being vitamin D deficient, 36.1% took vitamin D supplements. An important caveat is that we neither had information as to when the pregnant women included in our study started supplementing nor the level of intake compliance. It appears that despite the use of vitamin D supplements, certain women might still be at risk of vitamin D deficiency. Indeed, other modifiable (BMI) and non-modifiable (genetics) factors are thought to influence the effect of vitamin D supplementation. A systematic review revealed that a significant percentage of serum 25-hydroxyvitamin level variation is explained by the vitamin D intake per kg of body weight [44]. In our study, among the 55 deficient women in group 4 and 5, 41.8% had a BMI above 25 kg/m^2^ at enrolment. Interestingly, even though not at a significant level, higher BMI at enrolment was a predictor of deficiency. It is thought that an increased vitamin D deposition in fat tissues potentially leading to decreased circulating levels [45].

The second objective of the present research was to establish a model that is able to best explain low circulating vitamin D levels in pregnant women and might help to identify women at risk of vitamin D deficiency in clinical settings. Women’s country of origin, smoking status and use of vitamin D supplementation were recognized upon univariable and multivariable logistic regression analyses as significant determinants of vitamin D deficiency in this Swiss sample. BMI at enrolment, even though not significant, also seemed to play a role in explaining low 25(OH)D concentrations. However, these variables will be needed to be tested in an independent sample to determine test performance compared with circulating 25(OH)D. Finally and against the researchers’ expectations, seasonality was not a significant parameter which could be included in our model. We believe that the non-statistically differences are due to limited sample size for each season.

A limitation of our study is the low generalizability driven by the small sample size and self-selection of study participants. However, more than two-thirds of women approached by the recruiting physicians participated in this study. Additionally, only a small proportion of the women included in the study had a Fitzpatrick index of V. Also, skin colour assessment did not take into account information regarding recent skin tanning associated to a fresh return from vacation in the sun or use of tanning booths. Furthermore, since women were asked at their visit to the clinic to participate in the study (and to actually participate in that visit), we kept our questionnaire rather short such that we did not assess dietary intake of vitamin D-containing foods in detail and neither did we collect information on the frequency of use and the dosage of vitamin D supplements.

## Conclusion

In conclusion, country of origin (correlated to skin colour), smoking habits, use of vitamin D supplements and to a certain extent BMI are important determinants of vitamin D deficiency in the first trimester of pregnancy in this group of women living in Switzerland. These criteria should be considered during the first prenatal consultation to ensure an early detection of deficiency and a prompt implementation of corrective measures when the deficiency is confirmed. The high prevalence of vitamin D deficiency observed in pregnant women in their first trimester suggests that Switzerland should adapt the prevention strategy currently in place and strengthen the focus on high-risk sub-groups.

## Additional files

Additional file 1: Questionnaire assessing variables and potential determinants of vitamin D deficiency in the study population. (DOCX 75 kb)

Additional file 2: **Table S1.** Comparison of Vitamin D levels in pregnant women in international studies. Table showing results of international studies that determined Vitamin D levels of pregnant women. (DOCX 29 kb)

## Abbreviations

AIC: Akaike information criterion
AUC: Area under the curve
BMI: Body mass index
CI: Confidence interval
DF: Degrees of freedom
LRT: Likelihood ratio test
LRχ^2^: Likelihood ratio Chi-square test
OR: Odds ratio
Q: Quartile
ROC: Receiver operator characteristic
USZ: University Hospital Zurich
