# Supplementary material for: Vitamin D status and its determinants in healthy pregnant women living in Switzerland in the first trimester of pregnancy
Source: BMC Pregnancy Childbirth. 2019 Jan 8;19:10. doi: 10.1186/s12884-018-2150-1 (PMC6323787; doi:10.1186/s12884-018-2150-1)
Supplement: Supplementary file 1 — Questionnaire assessing variables and potential determinants of vitamin D deficiency in the study population. (DOCX 75 kb) [file 12884_2018_2150_MOESM1_ESM.docx]

**Questionnaire**

Study title:

A cross-sectional study investigating vitamin D status, parathyroid hormone and (epi)genetic determinants in pregnant women and their newborns

Teilnehmerin

_ Nr:_________________________

Einwilligungserklärung unterschrieben am ______________

Zuständiger Arzt / zuständige Ärztin ________________________

Geprüft von __________________________ am ________________

| Date of birth: | I__I__I / I__I__I / I__I__I  D M Y | | Study number: | | __________________ |
| --- | --- | --- | --- | --- | --- |
| Country of origin:  In which country:  was your mother born?  was your father born? | ____________________  ____________________  ____________________ | | Nationality: | | __________________ |
| What is your highest educational degree? | _________________ | | What is your husband’s / partner’s highest educational degree? | | _________________  Not applicable |
| Have you ever smoked? | 1)  Yes  2)  No | | Do you smoke now? | | 1)  Yes  2)  No |
| *Show the participant the scale and ask:*  Which face best describes your skin color?  Answer of the interviewer: ____ (please indicate the participant’s skin type here)  *(If the participant cannot decide between 2 options, the following additional questions can be asked:*  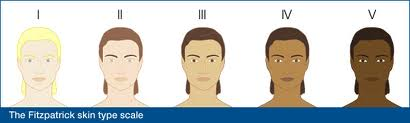 | | | | | |
| *Imagine a nice day in early summer at 12:00 noon.*  *What happens if your skin is untanned and you then lie out in the sun for 45-60 minutes?*  *Mark an answer. If you are uncertain, simply choose one option:*  *I)  painful sunburn 24 hours later, no tan 1 week later*  *II)  painful sunburn 24 hours later, slight tan 1 week later*  *III)  some sunburn 24 hours later, moderate tan 1 week later*  *IV)  no sunburn 24 hours later and tanned 1 week later*  *V)  My underlying skin color is brown/black, no sunburn, tanned 1 week later* | | | | | |
| On average, how many days a week are you usually outdoors between 10 a.m. and 4 p.m.?  In summer  1) I__I days < 1 hr  2) I__I days 1-2 hr  3) I__I days >2 hr | | On average, how many days a week are you usually outdoors between 10 a.m. and 4 p.m.?  In winter  1) I__I days < 1 hr  2) I__I days 1-2 hr  3) I__I days >2 hr | | | |
| On average, how many days a week were you outdoors **six months ago** between 10 a.m. and 4 p.m.?  1) I__I days < 1 hr  2) I__I days 1-2 hr  3) I__I days >2 hr | | | | | |
| In the summer, how often have you protected yourself from the sun (e.g. sunscreen, long-sleeved t-shirt or pants, long skirt)?  1)  never  2)  sometimes  3)  always | | | | | |
| On average, how many times per week do you eat these types of fish: ***Salmon, herring, mackerel, sardines, tuna***? | | | | ____ times per week | |
| Do you take vitamin supplements?   \|  \| Yes/No \| Brand name \| Daily \| Weekly \| Occasionally \| \| --- \| --- \| --- \| --- \| --- \| --- \| \| Vitamin D \|  \|  \|  \|  \|  \| \| Iron \|  \|  \|  \|  \|  \| \| Folate \|  \|  \|  \|  \|  \| \| Multivitamin \|  \|  \|  \|  \|  \| | | | | | |
