# Supplementary material for: Vitamin D status and its determinants in healthy pregnant women living in Switzerland in the first trimester of pregnancy
Source: BMC Pregnancy Childbirth. 2019 Jan 8;19:10. doi: 10.1186/s12884-018-2150-1 (PMC6323787; doi:10.1186/s12884-018-2150-1)
Supplement: Supplementary file 2 — Table S1. Comparison of Vitamin D levels in pregnant women in international studies. Table showing results of international studies that determined Vitamin D levels of pregnant women. (DOCX 29 kb) [file 12884_2018_2150_MOESM2_ESM.docx]

Table S1: Comparison of Vitamin D levels in pregnant women in international studies.
